# Extraction and Characterization of Cellulose Nanocrystals from Tea Leaf Waste Fibers

**DOI:** 10.3390/polym9110588

**Published:** 2017-11-07

**Authors:** Nur Hayati Abdul Rahman, Buong Woei Chieng, Nor Azowa Ibrahim, Norizah Abdul Rahman

**Affiliations:** 1 Department of Chemistry, Faculty of Science, Universiti Putra Malaysia, 43400 UPM Serdang, Malaysia; yatierahman08@gmail.com (N.H.A.R.); a_norizah@upm.edu.my (N.A.R.); 2 Materials Processing and Technology Laboratory, Institute of Advanced Technology, Universiti Putra Malaysia, 43400 UPM Serdang, Malaysia

**Keywords:** tea leaf waste fibers, acid hydrolysis, cellulose nanocrystals

## Abstract

The aim was to explore the utilization of tea leaf waste fibers (TLWF) as a source for the production of cellulose nanocrystals (CNC). TLWF was first treated with alkaline, followed by bleaching before being hydrolyzed with concentrated sulfuric acid. The materials attained after each step of chemical treatments were characterized and their chemical compositions were studied. The structure analysis was examined by Fourier transform infrared (FTIR) spectroscopy and X-ray diffraction (XRD). From FTIR analysis, two peaks at 1716 and 1207 cm^−1^—which represent C=O stretching and C–O stretching, respectively—disappeared in the spectra after the alkaline and bleaching treatments indicated that hemicellulose and lignin were almost entirely discarded from the fiber. The surface morphology of TLWF before and after chemical treatments was investigated by scanning electron microscopy (SEM) while the dimension of CNC was determined by transmission electron microscopy (TEM). The extraction of CNC increased the surface roughness and the crystallinity index of fiber from 41.5% to 83.1%. Morphological characterization from TEM revealed the appearance of needle-like shaped CNCs with average diameter of 7.97 nm. The promising results from all the analyses justify TLWF as a principal source of natural materials which can produce CNC.

## 1. Introduction

Studies about natural fibers among researchers have increased in the last few decades, especially as reinforcement filler in composite materials [1,2,3,4,5]. Natural fibers are widely used for applications in textiles, paper manufacturing, civil engineering, and bioenergy fields because of their attractive features such as lower density, greater deformability, availability, low cost, renewability, biodegradability, and lower abrasiveness compared with man-made fibers [6,7].

The components of natural fibers are mainly consist of cellulose, hemicelluloses, lignin, and a small amount of extractive substances. Their constituents are depending on the fiber type, age, and origin (bast or stem, leaf, seed or fruit, grass or straw fibers) [8]. Cellulose is a long chain of natural hydrophilic polymer consisting of poly(1,4-β-d-anhydroglucopyranose) units that are widely distributed in wood plants (e.g., cotton, flax, and sisal), in some of the marine animals and in algae, bacteria, fungi, invertebrates, and even amoeba (e.g., *Dictyostelium discoideum*) [9]. These units consist of three hydroxyl functional groups that allow cellulose to form strong hydrogen bonds and can be used as a starting material to produce cellulose nanocrystals (CNC). The other parts of natural fibers are hemicellulose and lignin. Hemicellulose is made up of a branched of heterogeneous polysaccharide polymer that consists of five groups of sugar types which are glucose, xylose, arabinose, galactose, and mannose. Besides that, hemicellulose has an amorphous nature and its molecular weight is lower than cellulose because it is partially water-soluble [10]. Whereas, lignin is a highly built up of three types of phenylpropane units which are mostly aromatic and amorphous, but have less absorption of water than other natural fiber components.

Although hemicellulose and lignin are both amorphous polymers in nature, cellulose is a semicrystalline polymer. There are two different bonding types between carbohydrate groups and lignin which are ester-type (the linkage is between the hydroxyl group of lignin and the carboxyl of uranic acid in hemicellulose) and ether-type (the linkage is between the hydroxyl of lignin and those of carbohydrates). The first linkage has a very poor alkaline resistance compared with the second linkage [11].

The tea leaf or its binomial name, *Camellia sinensis*, is a small evergreen shrub about three to six feet tall native to Asia. According to the Food and Agriculture Organization of the United Nations (FAO)’s statistics, there is increasing in world tea production to about 5.07 million tons in year 2013 [12]. Tea leaf waste fiber (TLWF) is a waste product of tea leaf processing, extracted after drying and chopping off the leaves. In industrial production, an abundance of tea leaf waste fiber is produced from the extraction of tea for instant tea and beverage tea. Negative environmental effects caused by simply disposing of tea waste could be diminished by expanding uses for tea waste in industries as an inexpensive source of biomass. So, it is necessary to make good use of the TLWF to increase its residual value [13]. Although the uses of tea leaves are widely developed in industries, no study on the isolation or properties of the cellulose from TLWF has been conducted to date.

To extract CNC from lignocellulosic sources, different processes can be applied to remove components surrounding the crystalline cellulosic fiber. A well-known and effective treatment for the extraction of CNC is by hydrolysis method using sulphuric acid. The process of hydrolysis with sulphuric acid will initiate the negatively charged sulfate groups on the structure of native cellulose and thus hydrolyze the amorphous area, turning it into nanosized fibers [14]. Many researchers had used this technique to isolate CNC from various plant sources such as *Agave angustifolia* [15], *Phormium tenax* [16], banana pseudostems [17], coconut rusk [18], kenaf [19], oil palm mesocarp [20], etc. The objective of this research was to extract CNC from TLWF as an alternative to synthetic fibers to produce a sustainable composite material. The resultant material was then investigated to determine its physicochemical and structural properties.

## 2. Materials and Methods

### 2.1. Materials

TLWF was gathered from local tea mill in Cameron Highlands (Pahang, Malaysia). Sodium chlorite was purchased from ACROS ORGANICS (NJ, USA) and acetic acid glacial from SYSTERM (Kuala Lumpur, Malaysia). Sodium chloride, sodium hydroxide, potassium hydroxide, and sulphuric acid were purchased from R & M Chemicals (Selangor, Malaysia). All chemicals were used as received without further purification.

### 2.2. Preparation of Cellulose Fibers

Purification of cellulose was conducted through alkaline treatment by eliminating lignin and hemicellulose from raw TLWF. TLWF with a diameter smaller than 150 μm was prepared and then treated in 4 wt % NaOH solution at 80 °C under mechanical stirring. After 3 h, the fibers were filtered from NaOH solution and washed with distilled water for several times to remove an excess NaOH. This alkaline process was repeated three times. Next, the bleaching process was performed at 80 °C by stirring the alkaline-treated fibers in equal volume (100 mL) of aqueous chlorite (1.7% *w/v* NaClO_2_), acetate buffer (2.7 g NaOH and 7.5 mL of glacial acetic acid in 100 mL of distilled water), and distilled water. After 4 h, the fibers were filtered and washed. This treatment was repeated until the color of fibers changed to white. Lastly, the fibers were air-dried at room temperature [21].

### 2.3. Extraction of Cellulose Nanocrystals

CNC was extracted from cellulose via acid hydrolysis. This step was conducted at 45 °C under mechanical stirring using 65 wt % sulphuric acid, H_2_SO_4_ (pre-heated) for 45 min. The reaction was stopped by adding the cold distilled water (4 °C) into hydrolyzed solution. The centrifugation process used to remove the excess of sulphuric acid from the sample was operated for 10 min at 9500 rpm and 10 °C. This cycle was carried out five times until the cloudy suspension was obtained. Dialysis process of suspension against distilled water was conducted at room temperature until a constant pH was obtained. Ultrasonic analysis was then performed to homogenize the CNC. The resulting suspension was kept in the refrigerator for further characterizations.

### 2.4. Characterizations

#### 2.4.1. Determination of Chemical Composition Analysis

The chemical composition of raw TLWF and treated TLWFs was studied. In order to determine the holocellulose content (i.e., cellulose and hemicellulose), raw TLWF was treated with acidified aqueous sodium chlorite (NaClO_2_) to remove the lignin as previously reported in literature [22,23]. The fibers were soaked in solution of 5 wt % NaClO_2_ at 70 °C for 1 h with a weight ratio of 1:20 fiber-to-NaClO_2_ solution. Before that, the NaClO_2_ solution was acidified with sulfuric acid (H_2_SO_4_) solution until the pH reached 4. The residue of sample was then filtered from solution, washed using distilled water, and dried in an oven at 60 °C. Meanwhile, the composition of cellulose was evaluated by soaking the holocellulose for 24 h with 6 wt % of potassium hydroxide solution at room temperature. After that, the sample was filtered, washed with distilled water, and oven-dried at 60 °C until a constant weight was obtained. The hemicellulose content of the fibers is the difference between the values of holocellulose and cellulose. The lignin content was determined by immersing the raw TLWF for 1 h in 72 wt % H_2_SO_4_ solution at 30 °C according to Technical Association of the Pulp and Paper Industry TAPPI standard method T222. The mixture was diluted to 3% H_2_SO_4_ and then retained for 2 h in reflux. The insoluble solid residue obtained was filtered, washed using distilled water, and oven-dried at 60 °C until a consistent weight was achieved. The processes were repeated for the treated TLWFs.

#### 2.4.2. Fourier Transform Infrared (FTIR) Spectroscopy

The FTIR spectra were recorded on an attenuated total reflection Fourier transform infrared (ATR-FTIR) by Shimadzu IRTracer-100 FTIR Spectrophotometer (Tokyo, Japan) to examine the changes in functional groups induced by various treatments. The FTIR spectral analysis was performed within a range of 400–4000 cm^−1^ with resolution of 4 cm^−1^.

#### 2.4.3. X-ray Diffraction (XRD) Analysis

The crystallinity of all the samples was analyzed by using a Shimadzu XRD 6000 Diffractometer (Tokyo, Japan) with a nickel-filtered Cu Kα (λ = 0.1542 nm) beam performed at 30 kV and 30 mA. The samples were examined with a scanning rate of 2°/min at 25 °C within a 2θ range of 5° to 40°. The crystallinity index (CrI) was then calculated by an empirical method using the Equation (1), as stated by Segal [24]

(1) $$\mathrm{CrI}\;\left(\%\right)=\frac{\left(I_{002}-I_{\mathrm{am}}\right)}{I_{002}}\times 100$$

where *I*_002_ is the maximum intensity of crystalline region and *I*_am_ is the lowest intensity of amorphous region of the sample.

#### 2.4.4. Scanning Electron Microscopy (SEM)

SEM was used to study the effects of various treatments on the morphology of the samples. The SEM micrographs of raw TLWF, alkaline-treated TLWF, and bleached TLWF were recorded by using JEOL (Tokyo, Japan) JSM-6400 scanning electron microscope operating at 15 kV accelerating voltage. The samples were prepared by placing them on a metal holder and coating with gold using a Bio-rad (Hercules, CA, USA) coating system for 3 min to ensure good conductivity prior to analysis.

#### 2.4.5. Transmission Electron Microscopy (TEM)

TEM was used to determine the dimensions of the CNC by investigated the hydrolyzed suspension using JEOL (Tokyo, Japan) JEM-1011 transmission electron microscope. The sample was prepared by mounting one drop of a diluted suspension of CNC on a copper grid crusted with a thin carbon film. The sample was then allowed to dry at room temperature before being stained with a 2 wt % solution of uranyl acetate for 1–2 min and then air dried.

## 3. Results

### 3.1. Chemical Composition of Tea Leaf Waste Fiber

The chemical composition of TLWF at each stage of chemical treatments is shown in Table 1. The raw TLWF consisted of 16.2% of cellulose, 68.2% hemicellulose, and 18.8% lignin. In alkaline treatment, sodium hydroxide (NaOH) was found to be effective in eliminating the surface impurities, hemicellulose, and lignin from TLWF, as the amount of hemicellulose was decreased by more than half from 68.2 to 22.2 wt %, while the lignin content was reduced to from 18.8 to 5.5 wt %.

A further removal of hemicellulose and lignin contents was studied by conducting a bleaching treatment. As expected, a higher content of cellulose was found after the bleaching and the content value increased from 58.8 to 87.9 wt %. These results strongly show that the treatments were efficient in removing most of the hemicellulose and lignin, resulting in high cellulose content.

**Figure 1:**
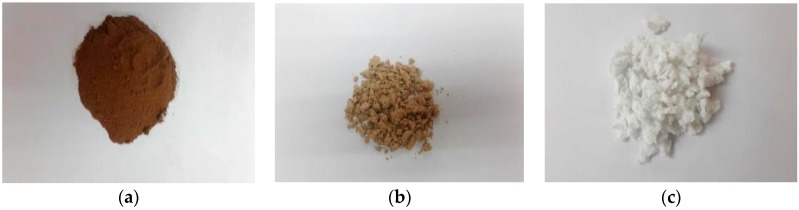
Photographs of (**a**) raw TLWF; (**b**) alkaline-treated TLWF; and (**c**) bleached TLWF.

Figure 1 shows the physical appearances of raw TLWF, alkaline-treated TLWF, and bleached TLWF. The color of the TLWF was changed as a result of the treatments. The color of alkaline-treated TLWF changed to light brown from brown which is the original color of raw TLWF, and then turned white after the bleaching process due to the removal of hemicellulose and lignin substances [25].

### 3.2. Fourier Transform Infrared (FTIR) Analysis

**Figure 2:**
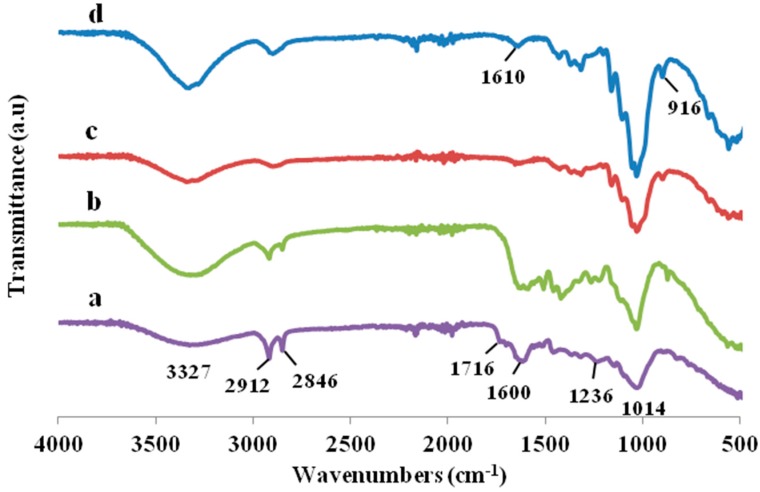
FTIR spectra of (a) raw TLWF; (b) alkaline-treated TLWF; (c) bleached TLWF; and (d) cellulose nanocrystals (CNC).

The FTIR was used to analyze the effects of alkaline and bleaching treatments on the functional groups, bonding types, and chemical constituents of the fibers. The spectra of raw TLWF and treated fibers were shown in Figure 2. A shoulder was observed at 1716 cm^−1^ for the raw TLWF is accredited to the C=O stretching vibration of the acetyl and uronic ester groups, from pectin, hemicellulose, or the ester linkage of carboxylic group of ferulic and *p*-coumaric acids of lignin and/or hemicelluloses [26]. Meanwhile, the peak at 1236 cm^−1^ in raw TLWF spectrum is attributed to the C–O stretching vibration of the acetyl or aryl groups in the lignin [27,28]. When the fibers are chemically treated, these two peaks slowly diminished in the spectra due to the elimination of hemicellulose and lignin during the treatments. Other researchers have also been reported the same results [29,30].

The spectra of raw TLWF and CNC revealed the same peaks in all wavenumbers but the only difference is the changes of peak intensities. All of the spectra showed a broad band region at 3400 to 3300 cm^−1^, which determined the free O–H stretching vibration of the OH group in cellulose molecules [31]. Whereas, all of the spectra exhibited around 2912 cm^−1^ indicated the characteristics of C–H stretching vibrations consist in cellulose components, interrelating to the lignin molecules [15]. The occurrence of reduction intensity at the absorption peak due to the removal of lignin composition in fibers shows that the alkaline treatment did not efficiently eliminate the lignin constituents, but bleaching treatment successfully removed the lignin.

The intensity of the absorbance peak at 1600 cm^−1^, which reflects the C=C stretching of aromatic rings in lignin was reduced after treatments indicated that the partial elimination of hemicellulose, lignin, and waxy substances had occurred [4,32]. Meanwhile, the peak at 1014 cm^−1^ is due to the C–O–C vibration of the pyranose ring skeletal [33]. A small peak at 916 cm^−1^ represented the glycosidic C_1_–H deformation, which is characteristic of β-glycosidic linkages between glucoses in cellulose. The intensity of these peaks increased upon treatments due to the increment of the percentage of cellulosic constituents, implying that the samples are more pure cellulose after alkaline and bleaching treatments. This result was supported by chemical composition analysis. The differences between the FTIR spectra of raw TLWF and CNC strongly prove that CNC was successfully isolated from TLWF by sulphuric acid hydrolysis.

### 3.3. X-ray Diffraction (XRD) Analysis

**Figure 3:**
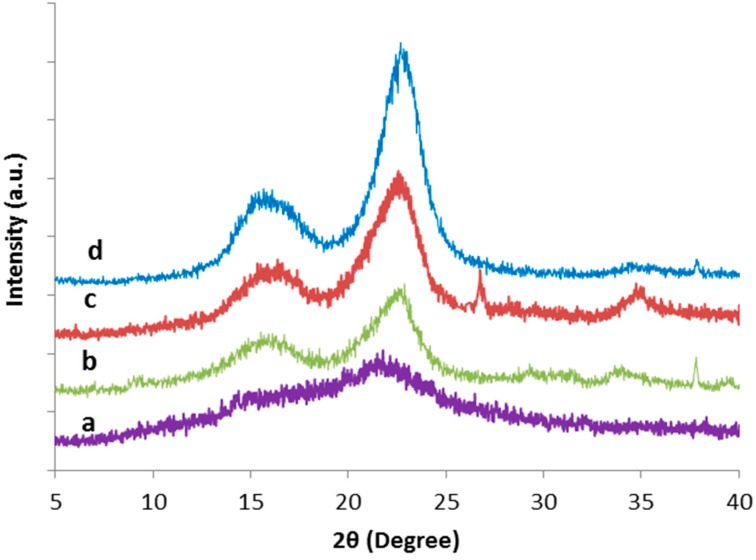
XRD diffractograms of (a) raw TLWF; (b) alkaline-treated TLWF; (c) bleached TLWF and; (d) CNC.

In nature, cellulose consists of both crystalline and amorphous regions whereas the hemicellulose and lignin are amorphous. The crystallinity index (CrI) of cellulose can be expressed as the ratio of the diffraction from a crystalline region to the total diffraction of the sample [33]. The XRD diffractograms for TLWF at different steps of chemical treatments and CNC obtained are shown in Figure 3, whereas the value of CrI was summarized in Table 2.

From the results, it was shown that raw TLWF has the lowest percentage value of CrI (41.5%), this is expected because it has a high content of amorphous regions in fiber. Upon treatments, the CrI of the fibers was increased from 41.5% to 74.4%. However, the alkaline treatment leads to greater improvement in CrI compared to bleaching treatment. The CrI of CNC further increased to 83.1% after the acid hydrolysis process. The increment in CrI indicates that the contaminants, waxiness, hemicellulose, and lignin substances were removed from the raw TLWF [23,34].

### 3.4. Scanning Electron Microscopy (SEM)

**Figure 4:**
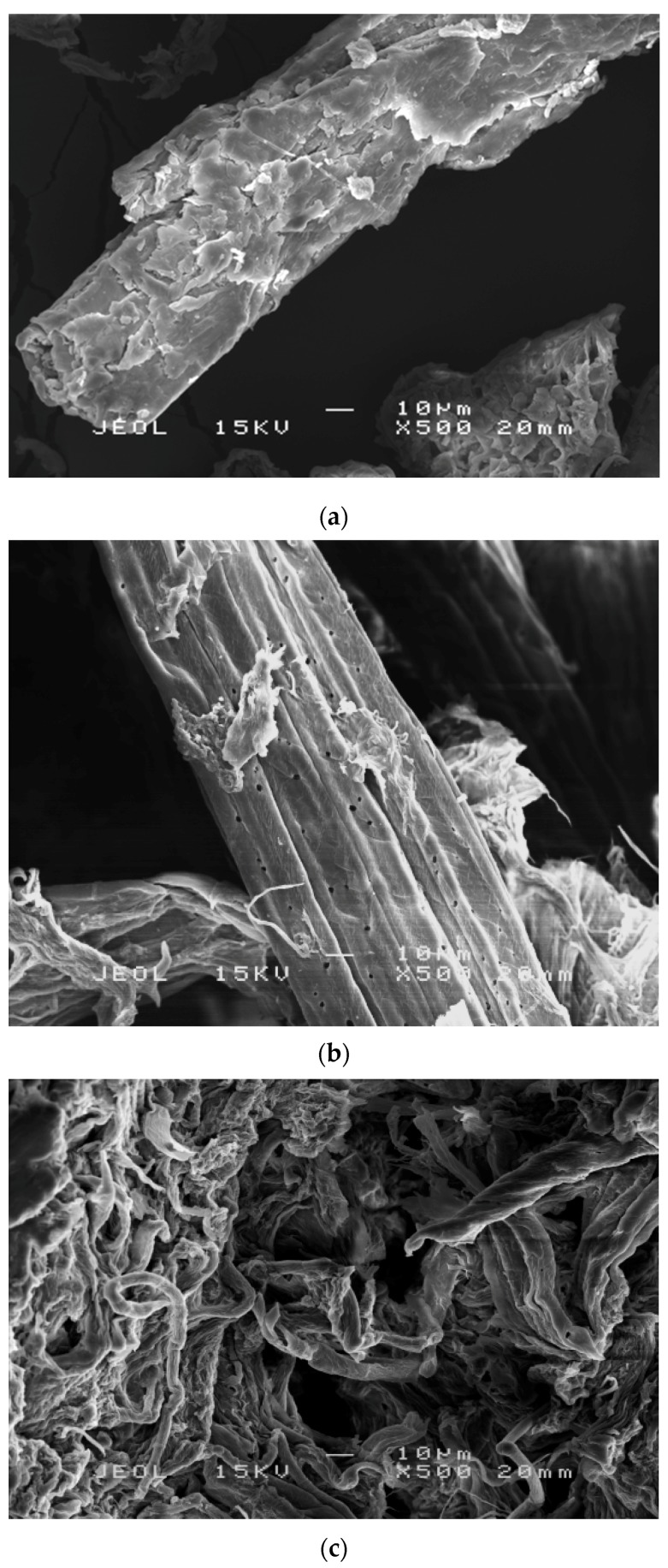
SEM micrographs of (**a**) raw TLWF; (**b**) alkaline-treated TLWF; and (**c**) bleached TLWF.

The morphology of TLWF surfaces at different stages of chemical treatment was determined by using SEM. Figure 4 shows that significant changes were observed on the fiber surface morphologies following the different chemical treatments. The changes are very important to figure out the interaction in between fibers and polymer matrix in the composites [15].

In Figure 4a, raw TLWF consists of bundles of continuous single cells that are bonded together by cement materials. The surface of raw TLWF was rough, irregular, and crusted with some contaminants such as hemicellulose, lignin, pectin, and waxy elements. However, after the alkaline-process, the surface morphology of TLWF appears relatively clean and porous can be seen as shown in Figure 4b. The surface of alkaline-treated TLWF appeared cleaner than raw TLWF because the impurities and extractives from the fibers were partially diminished. Hemicellulose is disintegrated and became water-soluble due to alkaline treatment process. Similar results have also been recorded in previous studies [29,30]. This phenomenon will help in the opening of the fiber bundles and defibrillation of the fibers.

In Figure 4c, the surface of bleached TLWF appeared even cleaner than alkaline-treated fiber, as more non-cellulosic materials or contaminates were eliminated. Thus, the isolation method of CNC is more susceptible because the fiber bundles were started to disintegrate into individual cells due to the elimination of lignin during the bleaching process [20].

### 3.5. Transmission Electron Microscopy (TEM)

**Figure 5:**
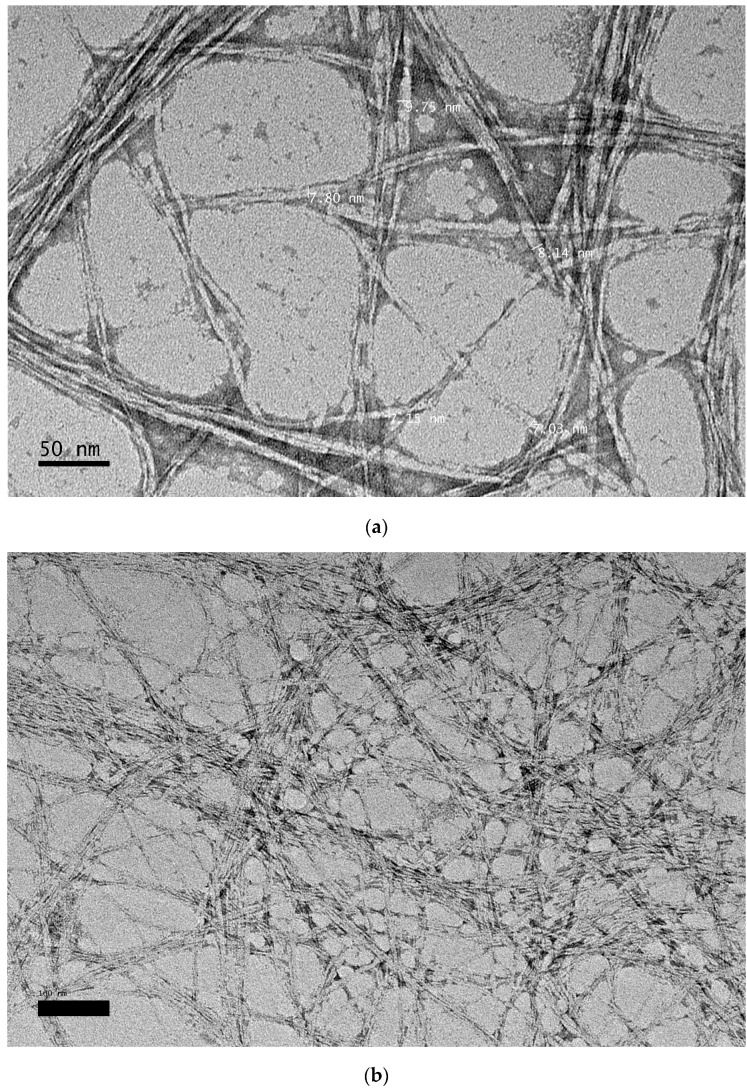
TEM micrograph of CNC extracted from TLWF at magnification of (**a**) 50,000× and (**b**) 25,000×.

TEM was used to examine the morphology and size of CNCs. The acid hydrolysis process allows the elimination of the amorphous area from cellulose fibrils by splitting the cellulose microfibrils into bunches of CNCs. These bundles contain individual fibrils of CNC. Figure 5 shows the TEM micrograph of CNCs with magnification of 50,000×. From the micrograph, the diameter of rod-like CNCs was determined and they had an average diameter of 7.97 nm with little agglomeration. The CNCs tend to agglomerate, probably due to surface ionic charge which had the crystallites stacked together as a result of the acid hydrolysis process [35]. The existence of the aggregates may also be a result of TEM sample preparation when the dispersing medium was removed [36].

## 4. Conclusions

The extraction of CNC from TLWF was successfully carried out by acid hydrolysis process after the fiber was treated to a two-step chemical process, an alkaline and bleaching treatment. The physicochemical characterization of CNC extracted from TLWF indicated good level of purity and high crystallinity. SEM images showed significant changes on the surface morphologies of the fibers. The fiber surfaces became smoother and eventually showed great reduction in diameter and size after acid hydrolysis. TEM observation of CNC showed that the CNCs had a rod-like shape and an average diameter of 7.97 nm. These findings proved that TLWF can be utilized to produce valuable CNCs which may be used in various industrial applications such as filler in polymer composites.

## Figures and Tables

**Table 1:** Chemical composition of raw TLWF and treated TLWFs.

Material	Cellulose (wt %)	Hemicellulose (wt %)	Lignin (wt %)
Raw TLWF	16.2	68.2	18.8
Alkaline-treated TLWF	58.8	22.2	5.5
Bleached TLWF	87.9	8.1	1.8

**Table 2:** Crystallinity index of TLWF at different stages of treatments.

Samples	2θ (Amorphous) (°)		2θ (002) (°)		CrI (%)
	Degree	Intensity (*I*_am_)	Degree	Intensity (*I*_002_)	
Raw TLWF	18.0	240	21.7	410	41.5
Alkaline-treated TLWF	18.9	144	22.8	440	67.3
Bleached TLWF	18.9	158	22.5	616	74.4
CNC	18.7	146	22.6	866	83.1
